# CircRNA GFRA1 promotes hepatocellular carcinoma progression by modulating the miR-498/NAP1L3 axis

**DOI:** 10.1038/s41598-020-79321-y

**Published:** 2021-01-11

**Authors:** Shuai Lv, Yingxia Li, Hanbing Ning, Meihui Zhang, Qiaoyu Jia, Xijuan Wang

**Affiliations:** 1 Department of Gastroenterology, The First Affiliated Hospital of Zhengzhou University, Zhengzhou, 450018 Henan Province China; 2 Department of Pediatrics, Henan Provincial People’s Hospital; Zhengzhou University People’s Hospital; Henan University People’s Hospital, No. 7, Weiwu Road, Zhengzhou, 450003 Henan Province China

**Keywords:** Cancer prevention, Metastasis, Cancer, Gastrointestinal cancer, Liver cancer

## Abstract

Circular RNAs (circRNAs) play essential roles in tumorigenesis and tumor progression. CircRNA GFRA1 (circGFRA1) was dysregulated in many cancer samples and acted as an independent marker for prediction of survivals in various cancer patients. However, the functions and molecular mechanisms of circGFRA1 in hepatocellular carcinoma (HCC) remain unclear. We collected 62 HCC tissues and normal adjacent tissues to evaluate the expression of circGFRA1 and the relationship between circGFRA1 expression and HCC patients’ survival. We carried out a list of characterization experiments to investigate the roles and underling mechanisms of circGFRA1 and miR-498 in HCC progressions. CircGFRA1 was greatly increased in HCC tissues and cells, and the over-expression of circGFRA1 was intimately related with the advanced clinical stage and poor survival of HCC patients. The expression of circGFRA1 was negatively correlated with the expression of miR-498, but a positive correlation was found between circGFRA1 and NAP1L3 expression in HCC tissues. Silencing circGFRA1 inhibited the growth and invasion of hepatocellular carcinoma. Moreover, miR-498 over-expression or NAP1L3 inhibition could abrogate the oncogene role of circGFRA1 in HCC in vivo. Our findings indicated that circGFRA1 contributed to HCC progression by modulating the miR-498/NAP1L3 axis in HCC.

## Introduction

Hepatocellular carcinoma (HCC) is recognized as the most primary malignancy of the liver, and the 3rd leading cause for cancer-related death all over the world^1,2^. As a severe health problem worldwide, many HCC patients are experiencing late diagnostics and bad prognosis due to the extremely high chances of metastasis and recurrence^3,4^. Only a small percentage of HCC patients have the opportunities of curative resection or transplantation^5^. Therefore, it is urged to identify potential biomarkers for the prediction of prognosis, as well as novel and useful targets to design a more robust therapeutic approach. In recent years, a large number of non-coding RNAs, such as micro RNAs (miRNAs) and long non-coding RNAs (lncRNAs), were reported to be dysregulated in HCC samples^6,7^. Here, we are determined to investigate the functional mechanisms of circular RNAs in HCC for the improvement of diagnosis and therapeutic approaches.

Circular RNAs (circRNAs) are newly discovered groups of endogenous non-coding RNAs that could regulate gene expression in mammals^8,9^. They have been identified to participate in cellular developmental processe^10,11^. Xiong et al. reported that circRNAs might act as a new type of potential biomarkers and therapeutic targets for hepatocellular carcinoma^12^. He et al. reported that circRNA GFRA1 could regulate neuronal cell survival and differentiation. Some studies also have indicated that GFRA1 has a role in the progression and metastasis of human cancers such as breast cancer^13^ and osteosarcoma^14^. However, for HCC, the dysregulation of GFRA1 remains to be elucidated.

It was widely accepted that GFRA1 could act as competitive endogenous RNAs to co-regulate each other by sponging microRNAs^13,15^. Many biologists have illustrated that circRNAs could work as miRNA sponges to contribute to the regulation of cancers^13^. MiRNAs are endogenous single-stranded with ~ 23 nucleotide RNAs that have an essential role in the development of human cancers^16^. MiR-498 was previously demonstrated to have tumor-suppressive effects in regulating cancer cell progressions in ovarian cancer^17^, colorectal cancer^18^ and lung cancer^19^. Considering the significant role of miR-498 in so many cancer types, we are encouraged to discover the role of miR-498 in HCC, as well as its potential interactions with circGFRA1.

The nucleosome assembly proteins (NAP) originated mammalian cells and was identified as a family of evolutionarily conserved histone chaperones^20^. NAPL3 has been illustrated to play essential roles in maintaining cell viability, especially in the formation and maintenance of the nervous system^21^. Besides, Kress et al. reported that NAPL3 promoted pre-mRNA splicing in budding yeast^22^. Motivated by its role in cellular biology, we aim to investigate the roles and inner associations among GFRA1, miR-498 and NAP1L3, as well as their functional mechanisms in HCC.

## Results

### CircGFRA1 was markedly over-expressed in HCC

**Figure 1:**
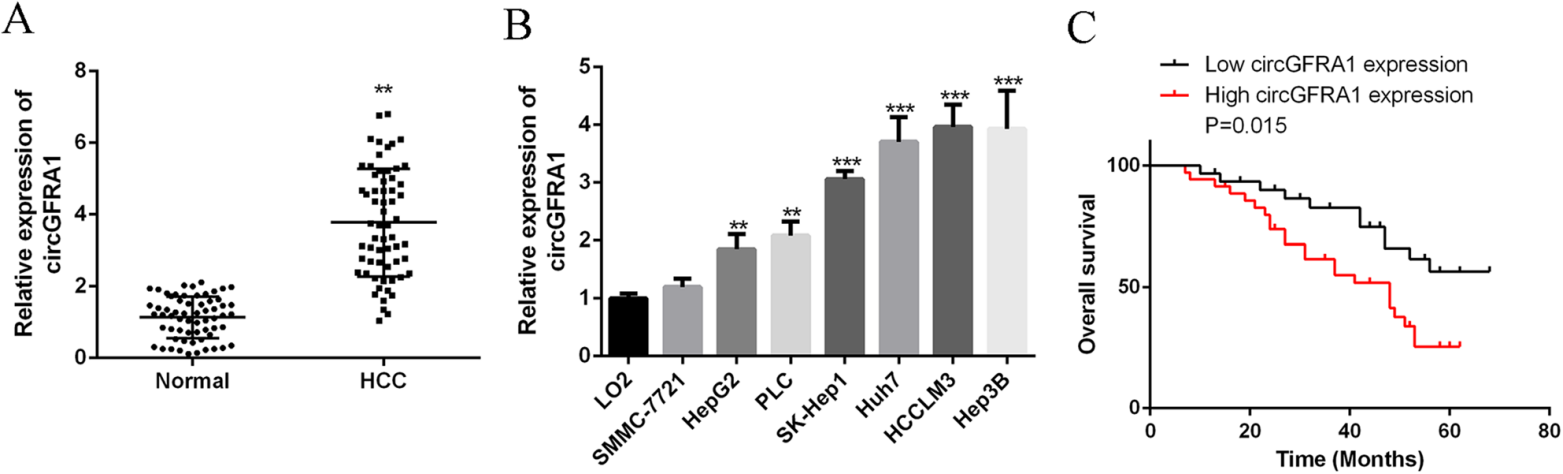
CircGFRA1 was markedly over-expressed in HCC. (**A**) qRT-PCR for circGFRA1 in HCC tissues and adjacent healthy tissues (n = 62). (**B**) mRNA of circGFRA1 in HCC cell lines and normal liver cell line by qRT-PCR. (**C**) Kaplan–Meier plots of HCC patients with low (n = 31) and high (n = 31) circGFRA1 expressions. Using median circGFRA1 values as the cutoff. **P < 0.01, ***P < 0.001.

To investigate the expression of circGFRA1 in HCC, qRT-PCR was used. Figure 1A showed that circGFRA1 was significantly over-expressed in HCC tissues compared with normal adjacent tissues (n = 62). Figure 1B demonstrated that circGFRA1 expression was significantly elevated in HCC cell lines of SMMC-7721, HepG2, PLC, SK-Hep1, Huh7, HCCLM3 and Hep3B compared with normal liver cell line. Moreover, as shown in Table 1, high circGFRA1 expression was markedly related to tumor size, intrahepatic metastasis, extrahepatic metastasis, BCLC stage and TNM stage. High circGFRA1 levels were also correlated with poor overall survival (Fig. 1C). The results revealed the oncogenic role of circGFRA1 in HCC.

**Table 1:** Correlation between GFRA1 expression and clinical pathological characteristic of HCC (n = 62).

Parameters	Group	n	GFRA1 expression		P-value
			High (n = 31)	Low (n = 31)	
Age (years)	60	28	13	15	0.625
	> 60	34	18	16	
Gender	Female	18	11	7	0.370
	Male	44	20	24	
Cirrhosis	Positive	41	23	18	0.121
	Negative	21	8	13	
AFP (ng/ml)	≤ 400	38	18	20	0.528
	> 400	24	13	11	
Tumor size (cm)	≥ 5	38	26	12	0.004**
	< 5	24	5	19	
Intrahepatic metastasis	Positive	21	14	7	0.011*
	Negative	41	17	24	
Extrahepatic metastasis	Positive	19	12	7	0.028*
	Negative	43	19	24	
BCLC stage	A	12	2	10	0.008**
	B + C	50	29	21	
TNM stage	I–II	22	3	19	0.002**
	III–IV	40	28	12	
Differetiation	Well-moderate	38	20	18	0.173
	Moderate to low–low	24	11	13	

X2 test was used to test the association between two categorical variables.

*P < 0.05, **P < 0.01.

### CircGFRA1 sponged miR-498

**Figure 2:**
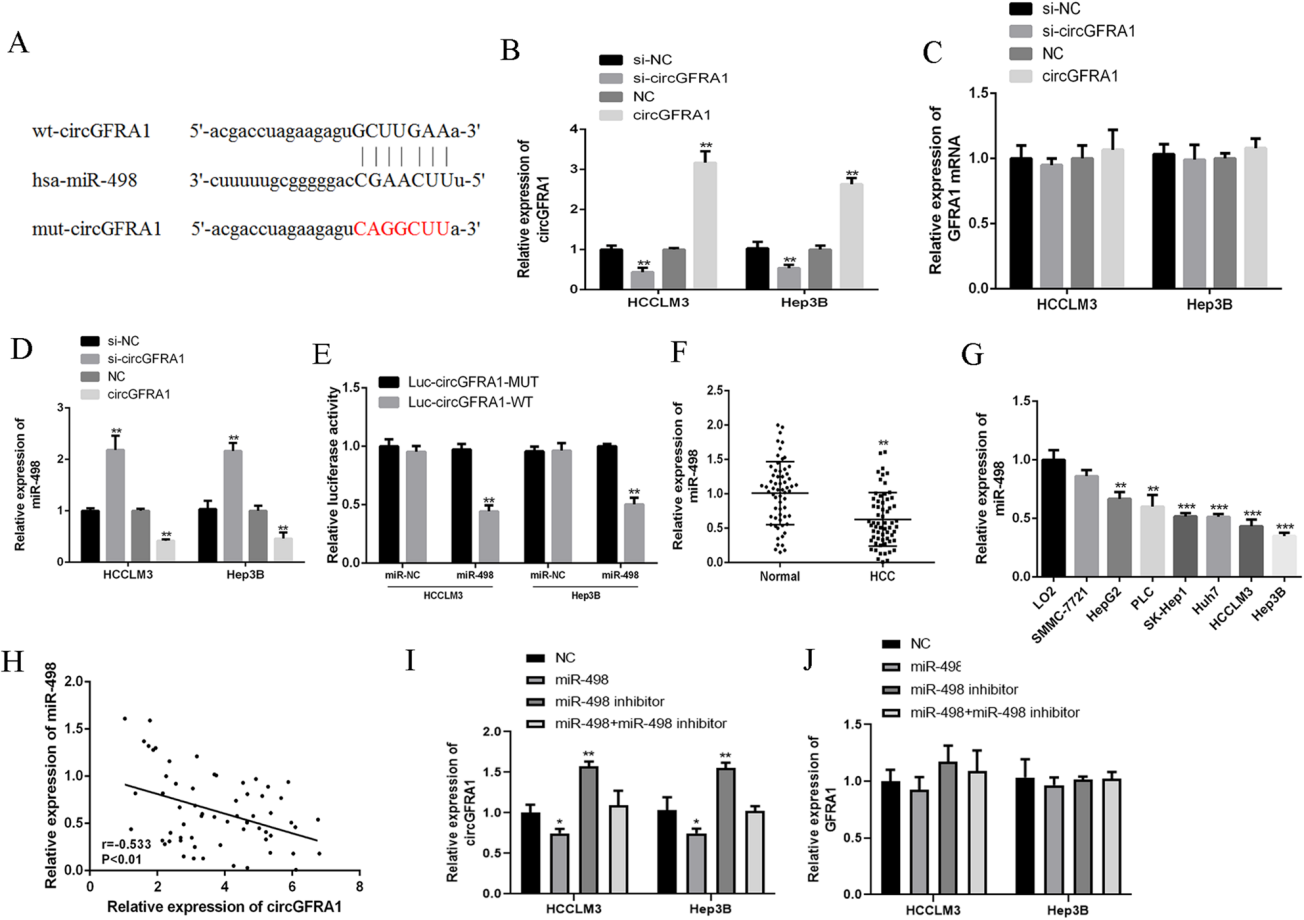
CircGFRA1 sponged miR-498 in HCC cell lines. (**A**) The common binding sequence of circGFRA1 and miR-498 by Starbase. (**B**–**D**) The expression of circGFRA1, GFRA1 mRNA and miR-498 by qRT-PCR in HCCLM3 and Hep3B cells transfected with si-circGFRA1 or circGFRA1. (**E**) Luciferase activity analysis in HCCLM3 and Hep3B cells co-transfected with miR-498 mimics and circGFRA1-WT or circGFRA1-MUT. (**F**) qRT-PCR for miR-498 in HCC tissues and normal tissues (n = 62). (**G**) qRT-PCR for miR-498 in HCC cell lines and normal liver cell line. (**H**) Pearson’s correlations between circGFRA1 and miR-498 in HCC tissues (n = 62) (r = -0.533, P < 0.01). (**I**) and (**J**) The expression of circGFRA1 and GFRA1 mRNA by qRT-PCR in HCCLM3 and Hep3B cells transfected with miR-498 mimics or inhibitor. *P < 0.05, **P < 0.01, ***P < 0.001.

Figure 2A searched the web tool Starbase and found that circGFRA1 might obtain shared binding sequences with miR-498. Figure 2B showed the qRT-PCR results and revealed that si-circGFRA1 effectively reduced circGFRA1 expression, but it did not affect its linear isoform GFRA1 mRNA (Fig. 2C). Similarly, as shown in Fig. 2C, circGFRA1 expression plasmid greatly elevated the expression of circGFRA1 and did not affect the expression of GFRA1 mRNA. In addition, Fig. 2D showed that circGFRA1 knockdown significantly promoted the expression of miR-498, but not circGFRA1 over-expression in HCCLM3 and Hep3B cells. From Fig. 2E, miR-498 significantly decreased the luciferase activity of the circGFRA1-WT, but did not change the luciferase activity of the circGFRA1-MUT. Moreover, Fig. 2F,G illustrated that miR-498 was greatly decreased in HCC tissues and cell lines compared with control. Figure 2H found a negative correlation between the expression of circGFRA1 and miR-498 in HCC tissues (r = − 0.533, P < 0.01). Figure 2I revealed that miR-498 effectively increased circGFRA1 expression, but it did not affect its linear isoform GFRA1 mRNA (Fig. 2J). Overall, it suggested that circGFRA1 might exert its functions by sponging miR-498.

### CircGFRA1 sequestered miR-498 and up-regulated NAP1L3 levels

**Figure 3:**
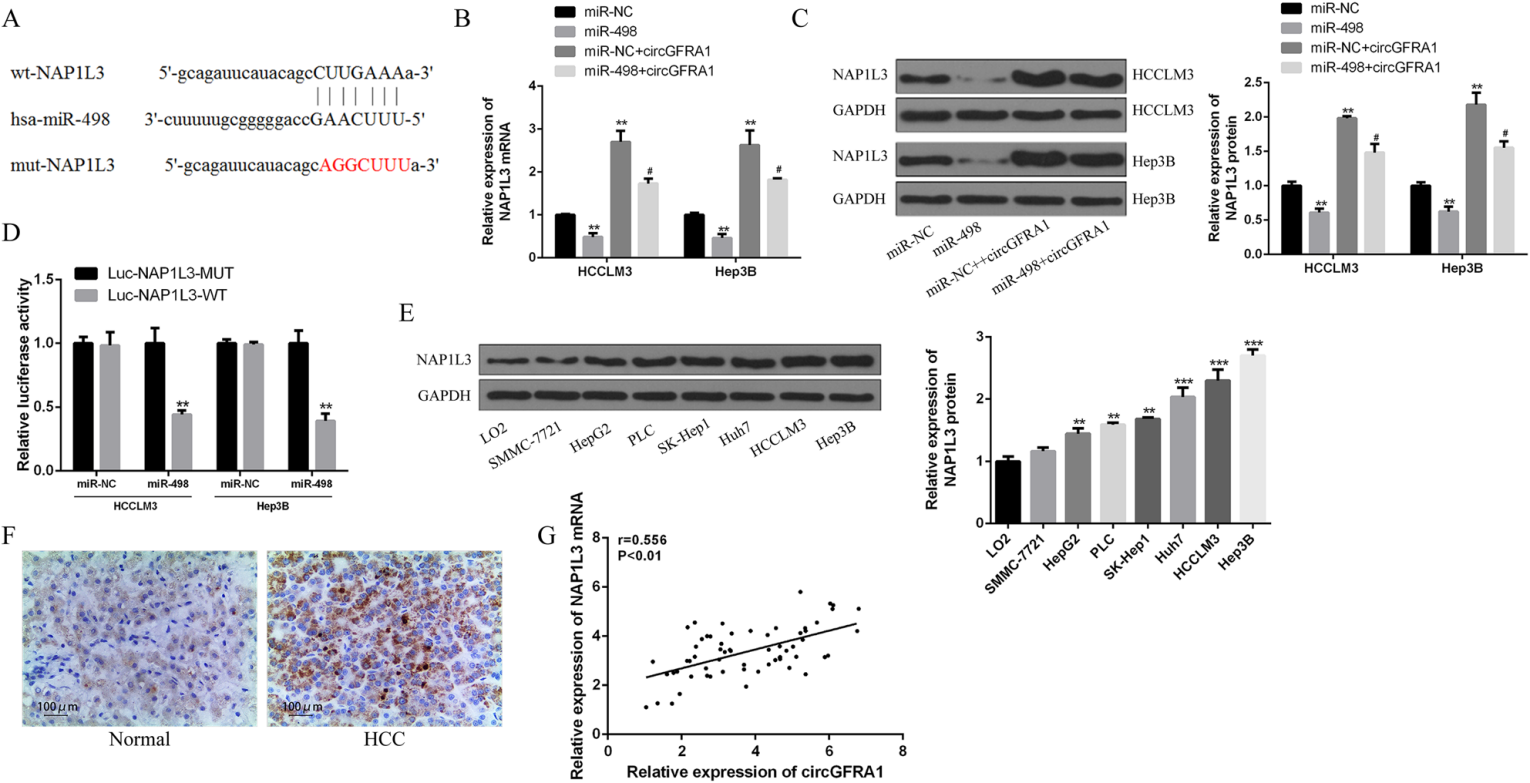
CircGFRA1 up-regulated NAP1L3 expression by sponging miR-498. (**A**) Putative binding sites of miR-498 and NAP1L3. (**B**,**C**) qRT-PCR and western blot for NAP1L3 mRNA and protein expression in HCCLM3 and Hep3B cells co-transfected with miR-498 mimics or miR-NC, and circGFRA1. (**D**) Luciferase activities in HCCLM3 and Hep3B cells co-transfected with miR-498 mimics and NAP1L3-WT or NAP1L3-MUT. (**E**) Western blot of NAP1L3 protein expression HCC cell lines and normal liver cell line. (**F**) IHC staining of NAP1L3 in HCC tissues and normal tissues. (**G**) Pearson’s correlation between circGFRA1 and NAP1L3 in HCC tissues (n = 62). *vs. control group, ^#^vs. miR-498 mimics group. **P < 0.01, ^#^P < 0.05.

Figure 3A showed the binding sites between miR-498 and NAP1L3. In Fig. 3B,C, we noticed that the mRNA and protein expression of NAP1L3 were significantly decreased in miR-498 in HCCLM3 and Hep3B. However, this effect was reversed by circGFRA1. Figure 3D found that miR-498 over-expression inhibited the luciferase activities of the NAP1L3-WT, instead of NAP1L3-MUT. Figure 3E,F demonstrate that NAP1L3 was markedly increased in HCC tissues and cell lines compared with control. According to Fig. 3G, we observed that circGFRA1 expression was positively related with NAP1L3 expression in HCC tissues (r = 0.556, P < 0.01). The above results revealed that circGFRA1 elevated NAP1L3 expression through sponging miR-498 in HCC (“Supplementary Information”).

### MiR-498 over-expression or NAP1L3 silencing effectively reversed circGFRA1-induced HCC progression

**Figure 4:**
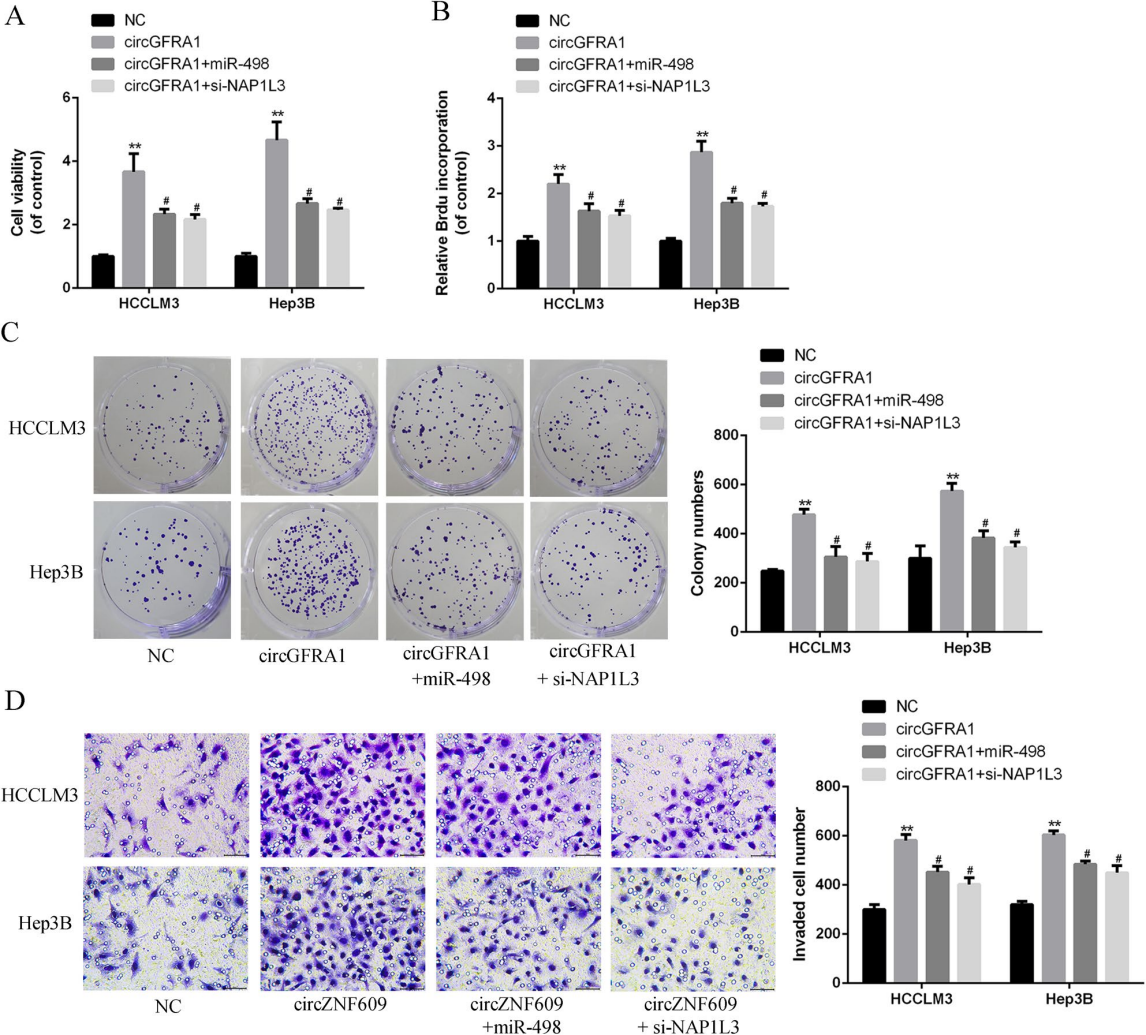
MiR-498 over-expression or NAP1L3 silencing effectively reversed circGFRA1-induced HCC progression. (**A**) CCK-8 assays of HCCLM3 and Hep3B cells co-transfected with circGFRA1 or control and miR-498 or si-NAP1L3. (**B**) Brdu incorporation assay of HCCLM3 and Hep3B cells co-transfected with circGFRA1 or control and miR-498 mimics or si-NAP1L3. (**C**) Colony formation assay of HCCLM3 and Hep3B cells co-transfected with circGFRA1 and miR-498 mimics or si-NAP1L3. (**D**) Transwell assay of HCCLM3 and Hep3B cells co-transfected with circGFRA1 or control vector, and miR-498 mimics or si-NAP1L3. Scale bar = 20 µm. *vs. control vector group, ^#^vs. circGFRA1 group. **P < 0.01, ^#^P < 0.05.

Based on the results from Fig. 4A–C, over-expression of circGFRA1 led to elevated HCC cell proliferation, and this effect was abrogated by miR-498 over-expression. Figure 4D also observed that miR-498 over-expression significantly reversed the circGFRA1-induced invasion rates. It was noted that circGFRA1 co-transfected with si-NAP1L3 had the same effect of co-transfection with miR-498 mimics on HCC malignant phenotypes.

### CircGFRA1 over-expression promoted NAP1L3-related signaling pathway in HCC cells

**Figure 5:**
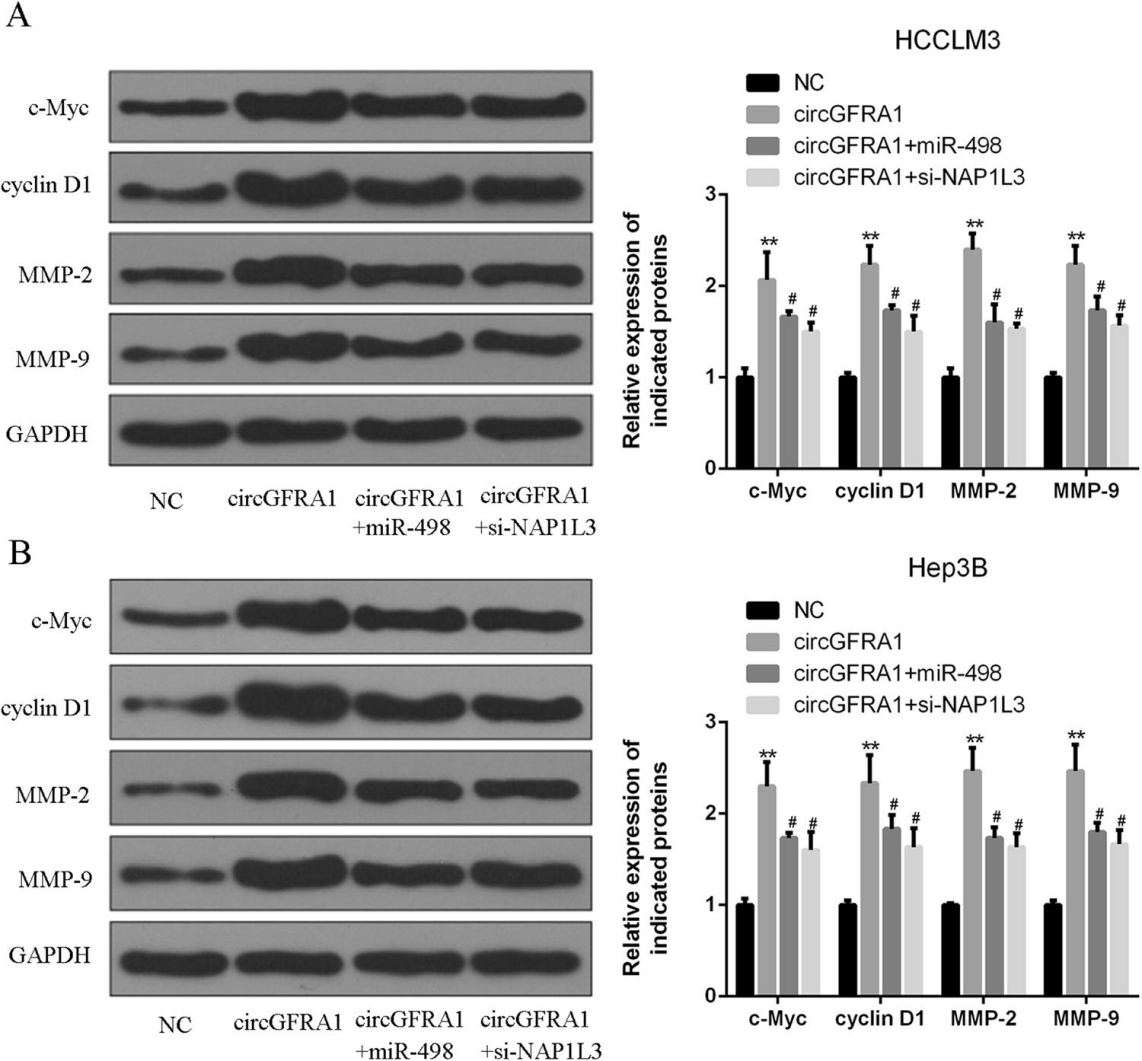
CircGFRA1 over-expression promoted NAP1L3-related signaling pathway in HCC cells. (**A**,**B**) Western blotting for c-Myc, cyclin D1, MMP-2, MMP-9 and GAPDH protein in HCCLM3 and Hep3B cells co-transfected with circGFRA1 or control and miR-498 mimics or si-NAP1L3. *vs. control vector group, ^#^vs. circGFRA1 group. **P < 0.01, ^#^P < 0.05.

Figure 5A,B observed that the expression of c-Myc and cyclin D1 (proliferation indicator) and MMP-2 and MMP-9 (invasion indicator) were strongly up-regulated in HCCLM3 and Hep3B cells transfected with circGFRA1 expression plasmid. However, these changes induced by circGFRA1 over-expression were partially abrogated by miR-489 mimics or si-NAP1L3 in HCC cells. Our data demonstrated that miR-498 over-expression or NAP1L3 knockdown could reverse circGFRA1-induced aggressive phenotypes of HCC cells.

### CircGFRA1 enhanced HCC progression in vivo by regulating the miR-498/NAP1L3 axis

**Figure 6:**
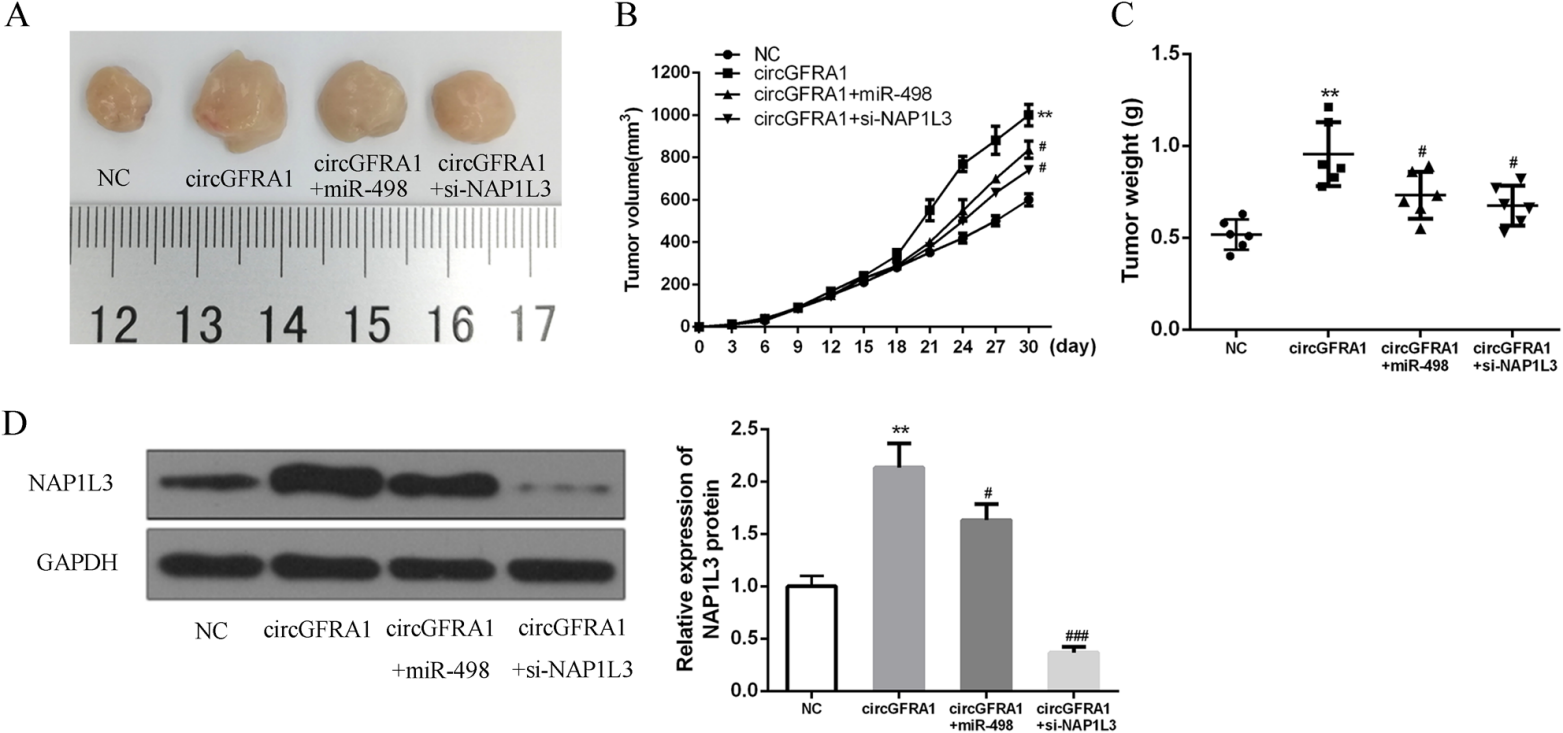
CircGFRA1 enhanced HCC progression in vivo by regulating the miR-498/NAP1L3 axis. (**A**) Subcutaneous tumor from 4 groups. (**B**) Tumor volumes of nude mice. (**C**) Tumor weight on mice at the 30th day. (**D**) The expression of NAP1L3 of NAP1L3 and GAPDH in cells transfected with NC, circGFRA1, circGFRA1 + miR-498, and circGFRA1 + si-NAP1L3 via using western blotting. (^#^P < 0.05 ^###^P < 0.01 vs circGFRA1, **P < 0.01 and ***P < 0.001 vs NC).

As shown in Fig. 6A, we found that the tumor volumes got 100 mm^3^ on 9th day. The group of circGFRA1 showed the largest tumor size, circGFRA + miR-498 had the 2nd largest tumor size, and circGFRA1 + si-NAP1L3 had a similar (a little smaller) size as the group of circGFRA1 + miR-498, when comparing with NC group. Figure 6B–D showed that circGFRA1 over-expression markedly elevated the tumor growth rate and tumor volume, accompanied by the upregulation of NAP1L3. Obviously, the promoted effects of circGFRA1 were relieved by miR-498 agomir and si-NAP1L3 in HCC cells. Our results indicated that circGFRA1 could effectively promote HCC progression by regulating the miR-498/NAP1L3 axis.

## Discussions

Although liver section and transplantation are available, hepatocellular carcinoma still ranks as the third leading cause for cancer-related death worldwide, especially in Asian^23,24^. Attributed to the immense opportunities of HCC cell metastasis, and tumor recurrence, HCC patients are experiencing a significant life risk even after tremendous treatments like surgeries, chemotherapies or targeted drugs^25,26^. Increasing studies have shown that various oncogenes are related to HCC metastasis, such as lncRNA PVT1^27^, lncRNA UCA1^28^ and circMTO1^29^. Therefore, it is quite important to reveal more therapeutic targets to improve the functional mechanisms and practical approaches for HCC patients.

There is growing evidence supporting that circRNAs are involved in the growth of colorectal^10^ and ovarian cancers^30^, as well as HCC^31^. CircGFRA1 has been implicated in the regulation of neuronal cell survival and differentiation^32^. Studies also have indicated that GFRA1 has a role in the progression and metastasis of human cancers such as breast cancer^13^ and osteosarcoma^14^. For instance, He et al. reported that circGFRA1 and GFRA1 act as ceRNAs in triple negative breast cancer by regulating miR-34a^13^. In 2015, Liu proposed that the downregulated expression of GFRa1 promoted HCC progression though Epithelial-to-Mesenchymal Transition^33^. Although this is a preliminary report, we still found its dysregulated expression in HCC samples. In our experiments, circGFRA1 was greatly elevated in HCC tissues and cell lines. High circGFRA1 expression was markedly related to tumor size, intrahepatic metastasis, extrahepatic metastasis, BCLC stage and TNM stage as well as poor overall survival. CircGFRA1 was dramatically over-expressed in HCC. It was in consistence with previous studies that circGFRA1 can be regarded as an oncogene in HCC.

It is well-known that lncRNAs could play as competitive endogenous RNAs to regulate other genes’ expression by sponging microRNAs^34^. Many findings have proved that circRNAs could function as miRNA sponges^35^. We found that circGFRA1 might directly sponge miR-498. The knockdown of circGFRA1 significantly promoted the expression of miR-498, and miR-498 significantly decreased the luciferase activity of the circGFRA1-WT. It was possible that there existed a negative correlation between the expression of circGFRA1 and miR-498 in HCC tissues. Our data further confirmed that circGFRA1 might exert its functions by sponging miR-498.

Some evidence indicates that NAP1L3 play essential roles in maintaining cell viability^36^. We noticed that NAP1L3 was markedly increased in HCC tissues and cell lines. The mRNA and protein expression of NAP1L3 were significantly decreased in miR-498 in HCC cell lines, and circGFRA1 expression was positively correlated with NAP1L3 expression in HCC tissues. CircGFRA1 elevated oncogene NAP1L3 expression by sponging miR-498 in HCC. In addition, we also found that miR-498 over-expression or NAP1L3 silencing effectively reversed circGFRA1-induced HCC progression. For the first time, we established the fact that circGFRA1 elevated NAP1L3 expression by acting as a sponge of miR-498 in HCC.

It’s reported that the knockdown of circGFRA1 inhibited proliferation and promoted apoptosis of triple-negative breast cancer cells^13^. From the western blotting results, the expression of proliferation indicators of c-Myc and cyclin D1 and invasion indicators of MMP-2 and MMP-9 were greatly elevated in HCC LM3 and Hep3B cells transfected with circGFRA1 expression plasmid. However, miR-489 mimics or si-NAP1L3 attenuated this effect. It was in consistent with previous studies, that miR-498 over-expression or NAP1L3 knockdown could reverse circGFRA1-induced aggressive phenotypes of HCC cells.

Le et al. have been found that NAP1L1 is a prognostic biomarker and contributes to doxorubicin chemotherapy resistance in human hepatocellular carcinoma^37^. NAP1L1 is an essential participant to the aggressive clinic pathologic features of HCC. We constructed the xenograft model based on nude mice and conducted in vivo experiments and compassion. We found that circGFRA1 over-expression markedly elevated the tumor growth rate and tumor volume, accompanied by the up-regulation of NAP1L3. However, the promoted effects of circGFRA1 were relieved by miR-498 agomir and si-NAP1L3 in HCC cells. As far as we know, we are the first to propose that circGFRA1 could effectively promote HCC progression by regulating the miR-498/NAP1L3 axis.

## Conclusion

These data suggest that circGFRA1 contributed to HCC progression by modulating the miR-498/NAP1L3 axis. Our findings may provide a potential therapeutic target for HCC.

## Methods

### Patients and tissue specimens

62 HCC tissues and healthy tissues were taken from Henan Provincial People’s Hospital. The experimental protocols were approved by the ethics committee of Henan Provincial People’s Hospital (No. HPPH201301HCC6#3), and has been performed in accordance with the Declaration of Helsinki. All patients signed the written informed consent. Patient inclusive criteria were in the following. They were pathologically diagnosed as HCC by two senior pathologists without adjunctive treatment before curative hepatectomy from 2013 to 2014. Patients were excluded if they had cholangiocarcinoma or other malignancy and incomplete clinical or prognostic data. All samples were collected in 15 min after removal from the body and immediately frozen in liquid nitrogen and stored at − 80 °C.

### Cell culture and transfection

Human HCC cell lines SMMC-7721, HepG2, PLC, SK-Hep1, Huh7, HCCLM3 and Hep3B, as well as normal liver cell line LO2 were purchased from the Cell Center of Shanghai Institutes for Biological Sciences. Hep3B cells were cultured in F12K (Gibco, USA), and the others were cultured in DMEM (Gibco, USA). Both mediums were added with 10% FBS (Gibco, USA) and 1% penicillin/streptomycin (Gibco, USA). Cells were incubated at 37 °C in a humidified atmosphere with 5% CO_2_. Si-circGFRA1, si-NAP1L3, miR-498 mimics, miR-498 agomir, and control (GenePharma, China) were transfected to 10^5^ cells via lipofectamine 2000 (Invitrogen, USA). Cells were collected at 24 h post-transfection for subsequent experiments. The sequence of si-circGFRA1 was 5′-AATAATGGTGAATAATGCGCA-3′. Human cDNA of circGFRA1 was cloned to a pcD-ciR vector to construct circGFRA1 over-expression plasmid (Geneseed Biotech., China), with Lipofectamine 2000 (Invitrogen, USA).

### QRT-PCR

Total RNA was isolated from 10^6^ cells via TRIzol kit (Invitrogen, USA). All RNA samples were digested with DNase I at 37 °C for 1 h to remove genomic DNA. QRT-PCRs were carried out by SYBR Premix Ex Taq II kit (Takara, Japan). GAPDH and U6 were regarded as controls. Three replicates were performed for each reaction. Gene expression levels were calculated using 2^−ΔΔCT^ method. The primer sequences were listed in the below.

- CircGFRA1 (divergent primer): forward: 5′-GTAGCTTATGCCGCGGCGG-3′
- CircGFRA1 (divergent primer) reverse: 5′-CAATCTTCGCAGTCAGGCG-3′
- GFRA1 (convergent primer): forward: 5′-CAACAGTGGTGAGGTTCGT-3′
- GFRA1 (convergent primer) reverse: 5′-CTGGTCAATGTGACGTGTGT-3′
- miR-498: forward: 5′-TCTGAGGTTTGGACCAATCGT-3′
- miR-498: reverse: 5′-TTCATCGCGCGGTAGGGCGG-3′
- NAP1L3: forward: 5′-GAGAAGAAGTGCTTCGCGAC-3′
- NAP1L3: reverse: 5′-TACTTGCGCCGAAGTTGGC-3′
- GAPDH: forward: 5′-ATGCGACCCACGGGAGAAT-3′
- GAPDH: reverse: 5′-AAAAAGGCTGCTTGTTGGAC-3′
- U6: forward: 5′-GCAGGGCTGTGATCTGTCGAC-3′
- U6: reverse: 5′-CCCCGACACCCCGGATTATTC-3′

### Animal studies

Sixteen 6-week-old female BALB/c nude mice (8 per group to provide a power of 90% for a significance level of 0.05 with a two-tailed t-test.) were classified to 2 teams. All the mice were subcutaneously administered with 10^6^ Hep3B cells for a xenograft model. At the 9th day, circGFRA1 over-expression plasmid, miR-498 agomir, si-NAP1L3 and negative control were intratumorally administered. After thirty days, we euthanized the mice. Tumor weights and volumes were measured and calculated by length × width^2^/2. The animals’ experiments were approved by the ethics committee of Henan Provincial People’s Hospital (No. HPPH201503HCC5A#522) and has been performed in accordance with the Basel Declaration.

### Cell proliferation assay

For CCK-8 assay, the transfected cells were seeded into 96-well plates (2000 cells/well) with 1 × 10^3^ cells/well. After one day, cell viability was detected by CCK-8 system (Dojindo, Japan). Briefly, each well was filled with 10 μl CCK-8 solution, and the plate was incubated at 37 °C for an hour in the dark. Signals were detected at 450 nm. For BrdU incorporation assay, the transfected cells were seeded in a 96-well plate (2000 cells/well). At 48 h post-transfection, cell proliferation was measured by BrdU cell proliferation assay kit (#5213S, Cell Signaling, USA). For colony formation assay, the transfected cells were seeded into 6-well plates at a density of 2000 cells/well and maintained in medium with 10% FBS. After 14 days, cell colonies could be directly observed, cells were fixed by 4% paraformaldehyde and stained by 0.1% crystal violet (Sigma, Germany) staining was conducted for 30 min. Colony-forming efficacy (%) was calculated through the ratio of colony number to cell seeding number. The colonies were recorded and measured.

### Transwell assay

24-well transwell chamber, without or with Matrigel (Corning, USA), was employed to detect hepatocellular carcinoma cell invasions. 5 × 10^4^ cells in non-serum culture medium were transferred to the upper chamber, and the lower chamber was filled with a culture medium with 20% FBS. The upper chamber was coated with Matrigel (BD Biosciences). Crystal violet (0.1%) was utilized to stain cells, and cells were observed under IX71 inverted microscope (Olympus, Tokyo, Japan). After one day, the migrated or invaded cells were fixed, stained and analyzed by using microscopy.

### Dual-luciferase reporter assays

The wild-type (WT) or mutated (MUT) sequences of circGFRA1 and NAP1L3 3′-UTR were cloned to a pmirGLO vector (Genearray Biotechnology, China). Cells were co-transfected with those reporter plasmids and miR-498-inhibitors or miR-498-mimics. Luciferase activies were assessed utilizing a dual-luciferase reporter assay system (Promega, USA) after 48 h incubation.

### Western blotting

HCC cells were lysed, and proteins were separated by electrophoresis (10% SDS-PAGE) and transferred to polyvinylidene difluoride (PVDF) membrane. After blocking for 2 h in 5% skim milk. They were incubated with antibodies of anti-NAP1L3 (1:1000, #ab158953, Abcam, UK), anti-Cyclin D1 (1:10,000, #ab134175, Abcam, UK), anti-c-Myc (1:1000, #ab32072, Abcam, UK), anti-MMP-2 (1:5000, #ab76424, Abcam, UK), anti-MMP-9 (1:1000, #ab32124, Abcam, UK) and anti-GAPDH (1:1000, #ab181602, Abcam, UK). The membranes were incubated at four Celsius for one night and treated by secondary antibody for 2 h at room temperature. The signals were detected by an ECL system (Bio-Rad., USA).

### Immunohistochemistry (IHC)

We fixed the tissues in 4% formalin, embedded them in paraffin and sectioned them (4 µm thickness). They were baked at 60 °C for 2 h and incubated with xylene for de-paraffinization and gradient ethanol. We carried out antigen retrieval and used 3% hydrogen peroxide to block endogenous peroxidase for twenty min. The sections were cured by goat serum to avoid non-specific staining. Then, they were treated with anti-NAP1L3 (1:1000, #ab158953, Abcam, UK) overnight at 4 °C. Primary Antibody Enhancer was used and incubated at room temperature for 20 min. Then, HRP Polymer (enzyme labeled second antibody) was added at room temperature for 30 min. At last, DAB was used to evaluate the results, and positive immune staining was measured by the proportion of positive cells.

### Statistical analysis

Data analyzing was carried out by GraphPad software 7.0 and SPSS19.0. All experiments were carried out in triplicate, and results represent the average of 3 independent experiments. Data were expressed as the mean value ± standard deviation. Student’s t-test (2 groups) and one-way ANOVA (> 2 groups) were utilized to analyze the differences. We employed Kaplan–Meier plots to evaluate the survival rates. Correlations were made by Pearson correlation. P < 0.05 was regarded as statistically significant.

## Supplementary Information

Supplementary Figures.

## Abbreviations

circRNAs: Circular RNAs
HCC: Hepatocellular carcinoma
WT: Wild-type
MUT: Mutated
NAP: Nucleosome assembly proteins
miRNA: Micro RNA
IHC: Immunohistochemistry
