# Supplementary material for: CircRNA GFRA1 promotes hepatocellular carcinoma progression by modulating the miR-498/NAP1L3 axis
Source: Sci Rep. 2021 Jan 11;11:386. doi: 10.1038/s41598-020-79321-y (PMC7801409; doi:10.1038/s41598-020-79321-y)

CircRNA GFRA1 promotes hepatocellular carcinoma progression by modulating the miR-498/NAP1L3 axis

Running title: CircGFRA1 promotes HCC progression via miR-498/NAP1L3 axis

Shuai Lv[1], Yingxia Li[1], Hanbing Ning[1], Meihui Zhang[2], Qiaoyu Jia[1], Xijuan Wang[2*]

1Department of gastroenterology, the First Affiliated Hospital of Zhengzhou University, Zhengzhou, Henan Province, 450018, China

2 Department of pediatrics, Henan Provincial People's Hospital, People's Hospital of Zhengzhou University, Zhengzhou, Henan Province, 450003, China

Correspondence author: * Xijuan Wang, Department of pediatrics, Henan Provincial People's Hospital, People's Hospital of Zhengzhou University, No.7, Weiwu Road, Zhengzhou, Henan Province, 450003, China Tel: +86-18703874060 Email: xijuanwang126@aliyun.com

1、Figure 3C: HCCLM3 cell： NAP1L3、GAPDH

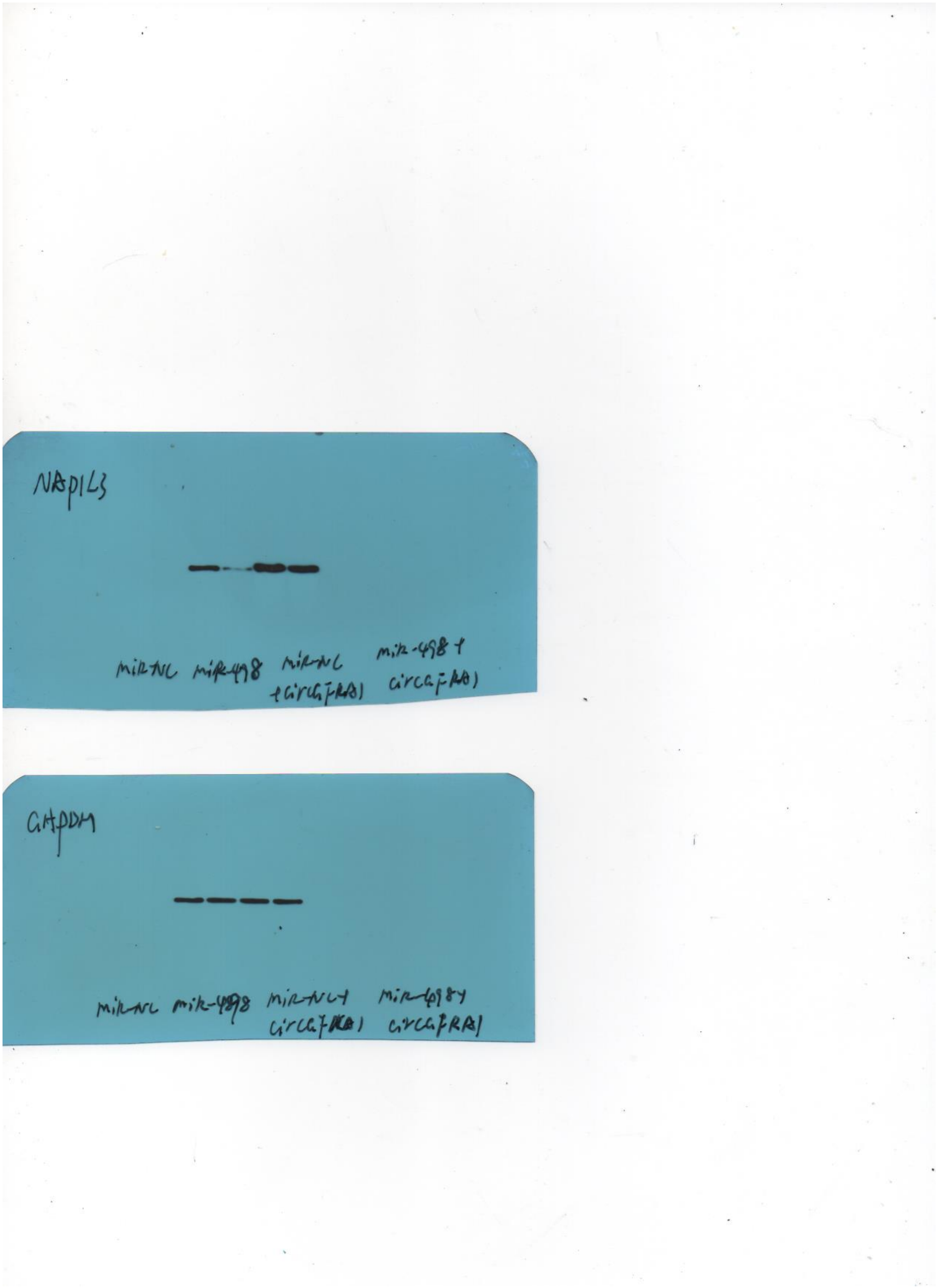

2、Figure 3C: HEP3B cell：NAP1L3、GAPDH

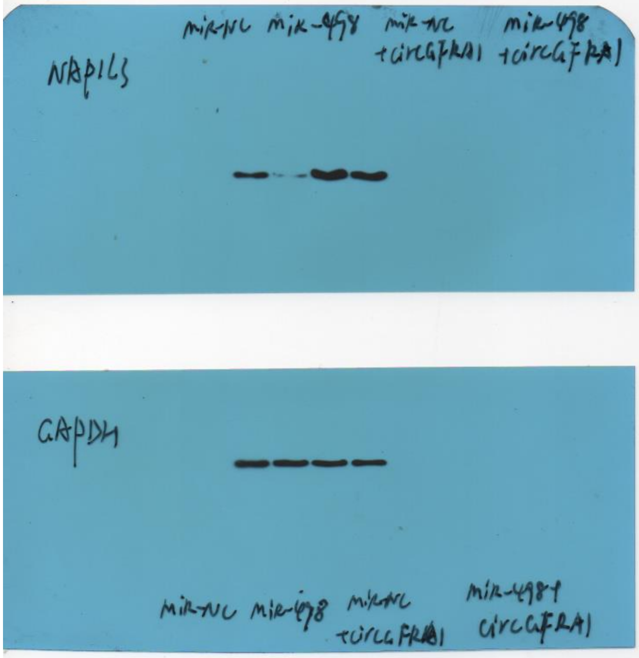

3、Figure 3E: NAP1L3、GAPDH

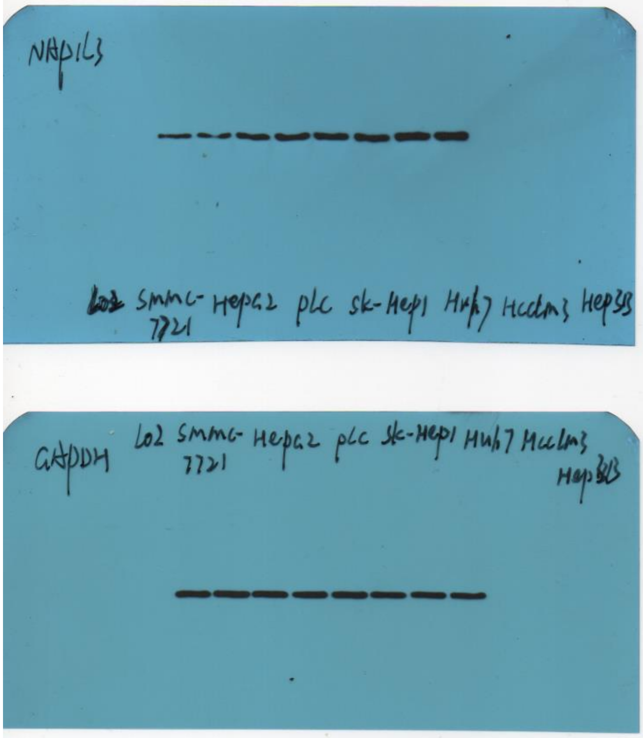

4、Figure 5A: HCCLM3 cell：c-Myc、cyclin D1、MMP-2、MMP-9、GAPDH

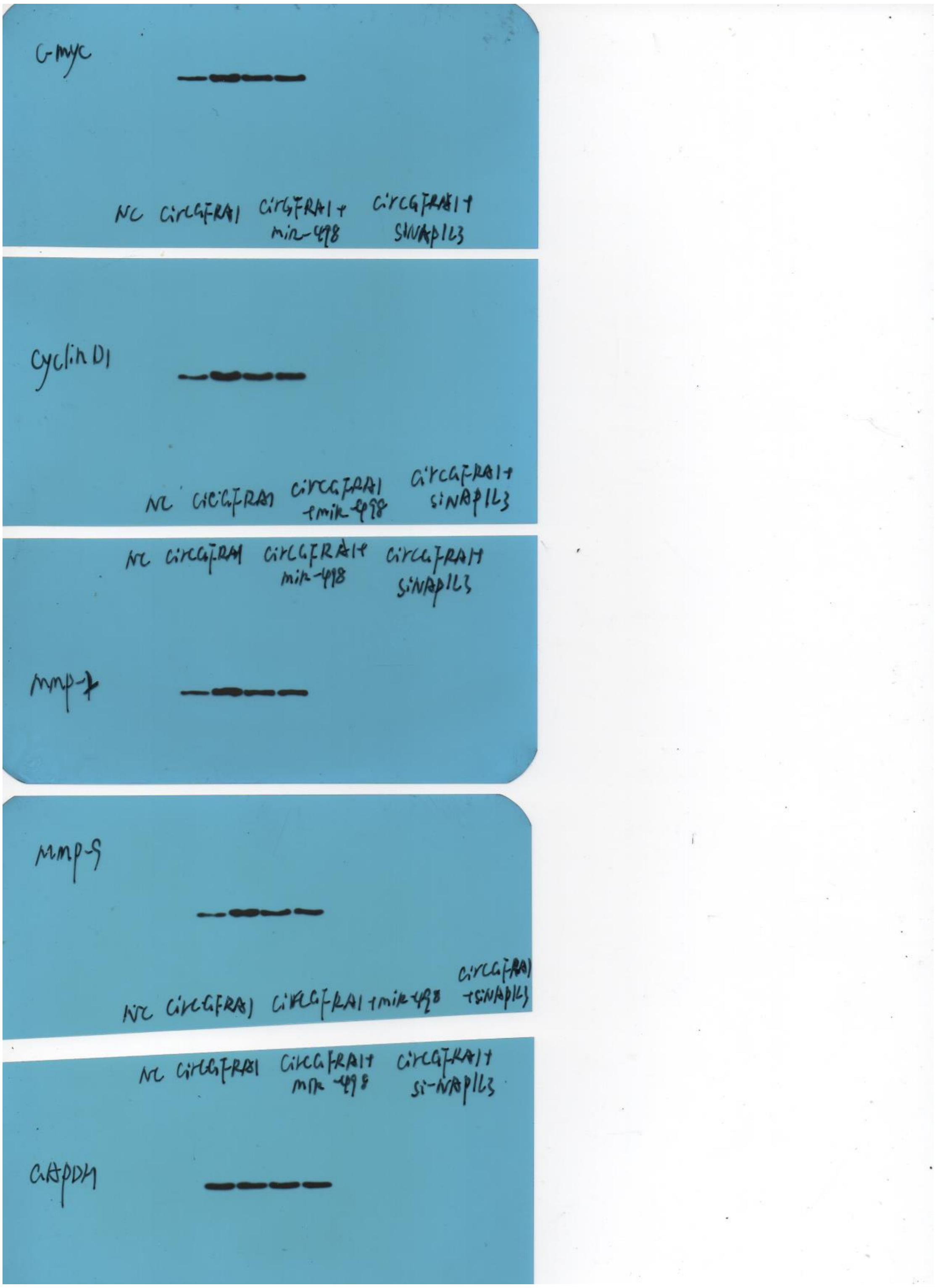

5、Figure 5B: HEP3B cell：NAP1L3、GAPDH

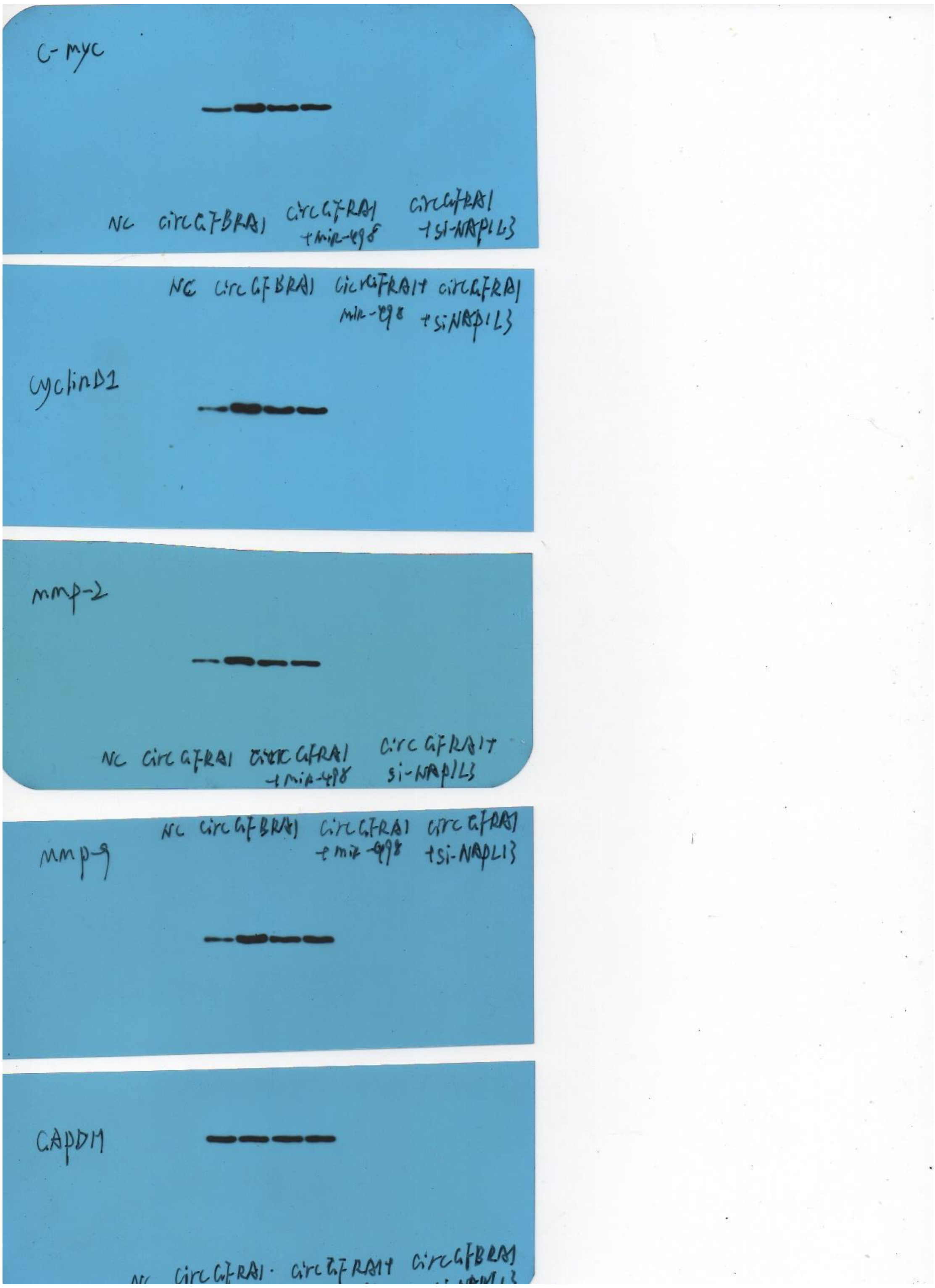

6. Figure 6D NAP1L3、GAPDH

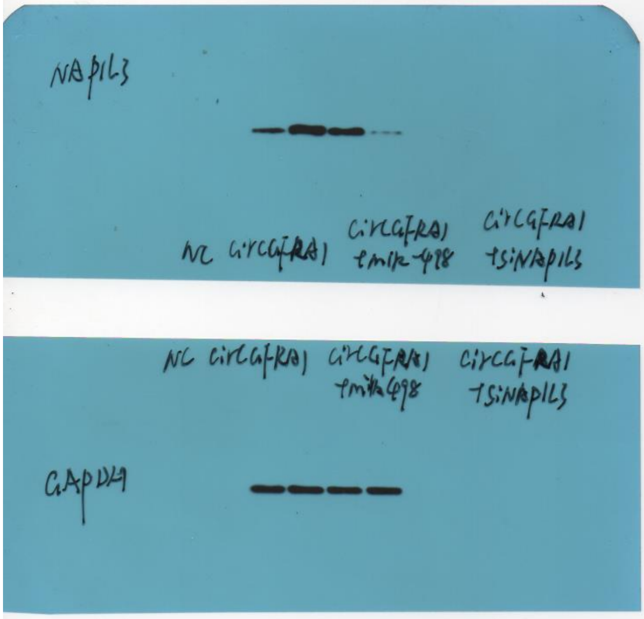

Supplement: Supplementary file 1 — Supplementary Figures. [file 41598_2020_79321_MOESM1_ESM.pdf]
